# Red cabbage extract immobilized in bacterial cellulose film as an eco-friendly sensor to monitor microbial contamination and gamma irradiation of stored cucumbers

**DOI:** 10.1007/s11274-024-04047-2

**Published:** 2024-07-02

**Authors:** Reham M. M. Abdelkader, Doaa A. Hamed, Ola M. Gomaa

**Affiliations:** Radiation Microbiology Department, National Center for Radiation Research and Technology (NCRRT), Egyptian Atomic Energy Authority (EAEA), Cairo, Egypt

**Keywords:** Anthocyanin, Gamma radiation, Smart packaging, Contamination, Cucumbers, Bacterial cellulose

## Abstract

The aim of the present study is to develop a pH-sensing biopolymer film based on the immobilization of red cabbage extract (RCE) within bacterial cellulose (BC) to detect contamination and gamma radiation exposure in cucumbers. The results obtained show a sensitivity to pH changes for RCE in its aqueous form and that incorporated within BC films (RCE-BC), both showed color change correlated to bacterial growth (R^2^ = 0.91), this was supported with increase in pH values from 2 to 12 (R^2^ = 0.98). RCE and RCE-BC exposure to gamma radiation (0, 2.5, 5, 10, 15, 20, 25 kGy) resulted in gradual decrease in color that was more evident in RCE aqueous samples. To sense bacterial contamination of cucumbers, the total count was followed at 0, 5, 10 and 15 days in cold storage conditions and was found to reach 9.13 and 5.47 log cfu/mL for non-irradiated and 2 kGy irradiated samples, respectively. The main isolates detected throughout this storage period were identified as *Pseudomonas fluorescens*, *Erwinia* sp. *Pantoea agglomerans* using matrix assisted laser desorption ionization–time of flight-ms (MALDI–TOF–MS). Bacterial growth in stored irradiated cucumbers was detected by color change within 5 and 10 days of storage, after which there was no evident change. This is very useful since contamination within the early days of storage cannot be sensed with the naked eye. This study is the first to highlight utilizing RCE and RCE-BC as eco-friendly pH-sensing indicator films for intelligent food packaging to detect both food contamination and gamma preservation for refrigerator stored cucumbers.

## Introduction

Food loss and waste (FLW) within agri-food supply chains is a persistent problem in the developing world (Kusumowardani et al. 2022). The loss in fruits and vegetables was estimated to 9.5 tons per week approximately (Dos Santos et al. 2020), resulting in financial losses of $ 510 million per year (Beretta et al. 2017). The microbial, enzymatic activities and continuous respiration that occur after harvesting fresh vegetables and fruits are mainly responsible for its deterioration. The core aspect of food safety is to detect contamination/spoilage rather than monitor it after it happens already. Therefore, there is a dire need to inform the consumers about food quality before eating or shopping in real-time monitoring (Yousefi et al. 2018; Sobhan et al. 2022). The design and development of biosensors and intelligent packaging has been recently perceived as an integral element to maintain high quality and safe produce.

Intelligent packaging (IP) is an innovative approach that has been implemented to monitor various condition changes in the packaged products, including food quality (freshness, spoilage, or ripeness), microbial activity (generation of organic acids, CO_2_, volatile nitrogenous substances, sulfur derivatives) or other properties (transport and storage history) that affect food quality (Lu et al. 2020). Despite the available IP systems, the number commercially used at stores is considerably small (Priyadarshi et al. 2021). The worldwide market for IP was estimated to be about $17.5 billion in 2019 and it is predicted to reach $251.6 billion by 2025 (Sobhan et al. 2021). The IP systems have many advantages because they can monitor the storage conditions, pH, temperature, shelf life and quality aspects of foods by using various sensitive sensors and indicators such as food quality indicators, food analysis sensors, storage sensors, cross contamination labels, and barcodes (Zhang et al. 2019). However, there are some limitations for the current methods, as they are expensive, time-consuming, offer low precision and not environmentally friendly (Merz et al. 2020). Additionally, these materials can lead to unnecessary waste as they are non-recyclable (Taherkhani et al. 2020). On the other hand, pH-sensitive visual indicators are perceived as practical intelligent packaging systems. They provide real-time information about the food freshness and quality by monitoring the microbial growth or initiation of certain chemical reactions. The mode of action of these indicators is based mainly on the detection of qualitative/quantitative changes in the concentration of one or more substances inside the packaging by changing their color in accordance with the changing pH of food products (Wu et al. 2022; Wang et al. 2023). A visual pH indicator is composed of two parts: a dye which recognizes the pH variations and a solid support matrix which immobilizes the dye (Khezerlou et al. 2023). Previously, synthetic dyes such as bromothymol blue, methyl red, bromocresol green and phenol red, as pH indicators for monitoring freshness of chicken breasts depending on pH changes (Rukchon et al. 2014). However, synthetic dyes hold potential toxicity and renders chemosynthetic indicators problematic to be used in food products (Zeng et al. 2019). Therefore, replacing these synthetic dyes by naturally pH indicators has been conducted in recent years. Anthocyanin is a natural pigment that is extracted from plants. It is widely distributed in nature, easy to obtain, nontoxic, safe for food contact, ecofriendly, cheap, sustainable, and biodegradable that make them a good choice to replace synthetic dyes (Halász et al. 2023). Anthocyanin pigment from red cabbage extract (*Brassica oleraceae*) is one of the common natural dyes that is used as pH- sensitive indicator and shows different color spectrum in a wide pH range. The anthocyanin extracted from red cabbage is low-cost because it is naturally abundant in comparison to other natural sources, it is water soluble and can be easily extracted. On the other hand, the solid support matrix, which is the second part of intelligent packaging systems, plays an integral role in pH indicators. The characteristics of polymeric matrix (synthetic/natural/biodegradable), their nature (hydrophilic/hydrophobic), the type of interactions with anthocyanins (chemical/physical) and their structural properties (porous/compact films) can significantly affect the diffusion and response of the anthocyanins at different pH values (Kossyvaki et al. 2022). Various polymeric and biopolymeric materials have been utilized as supporting matrices to immobilize the dyes such as chitosan-corn starch (Silva-Pereira et al. 2015), gelatin (Musso et al. 2017), cellulose nanofibers (Pourjavaher et al. 2017), starch-cellulose nanocomplexes (Ezati et al. 2019), starch-polyvinyl alcohol (Zhai et al. 2017), polysaccharides, lipids, and proteins (Mohamed et al. 2020). Bacterial cellulose (BC) is a biopolymer that is characterized with unique properties and several food and non-food applications. BC is synthesized during fermentation process of kombucha tea as a floating solid polymeric film at the air–liquid interface of the culture medium (Villarreal-Soto et al. 2019). It has attracted great attention due to its high purity (free of hemicellulose, lignin, and pectin), the ultrafine structure with distinct tunnel and pore network architecture and the presence of active hydroxyl groups (Choi et al. 2022). Its hydrophilicity, high sorption ability for liquids (97%), high crystallinity (60–80%) and non-toxicity stimulate its use in various applications. Some studies mentioned that BC like hydrogels and can be used as bio-packaging material because of its edibility and biodegradability feature (Jang et al. 2023).

pH sensitive indicators are considered real-time detectors of food spoilage of meat, chicken, fish, Shrimp, milk and cheese (Zhang et al. 2019, 2020; Lee et al. 2019; Weston et al. 2020; Pirsa et al. 2020; Gast et al. 2021). Other studies were found in the literature for using pH smart indicator in examination of vegetables and fruits freshness such as date fruit (Maftoonazad and Ramaswamy 2019), Mangoes (Dirpan et al. 2018) and Fresh-cut green bell pepper (Chen et al. 2018). However, none was reported for cucumbers. Fresh cucumber (*Cucumis sativus* L.) is considered one of the most essential economic crops universally especially in Egypt (El-Dkeshy et al. 2023). According to FAO, about 613 thousand tons of gherkins and cucumber are being produced in Egypt from twenty-seven thousand hectares in which eleven thousand tons were exported in 2020 (FAOSTAT 2020). However, cucumbers are known to be easily contaminated, therefore, gamma radiation can be used to preserve cucumbers and hence increase their shelf life and maintain its quality (Khalili et al. 2017) and at the same time maintain the color of the anthocyanin pigment post irradiation (Ito et al. 2019). It is important to ensure the accuracy of the absorbed doses for reaching the desired sterilization process of the irradiated food products for safe consumption. Radiochromic dosimeters are an easy and reliable way that can be used to monitor the irradiation process depending on the discoloration phenomena in which the radiochromic dosimeter is equivalent to the absorbed dose by the irradiated material. The use of a natural, eco-friendly, and safe pigment as a substitute for synthetic dyes is considered advantageous and feasible. Therefore, from this standpoint, the present work aims to prove that aqueous red cabbage extract (RCE) and RCE incorporated within BC films can be used as smart pH-sensing indicators to detect early cucumber contamination and identify if the produce was exposed to gamma irradiation or not.

## Materials and methods

### Sample collection

Commercially ripe fresh cucumbers samples were bought from a local vegetable market in Cairo. Plastic stretch film (saran wrap) used for packaging of cucumbers; samples were immediately stored in the refrigerator at 4 °C ± 1 throughout the period of study. Periodically, three samples were taken for microbiological, physiochemical analysis.

### Microbiological analysis

Total plate counts (TPCs) counts were determined during cucumbers storage period (0, 5, 10, 15 days) by the standard methods reported in APHA (1992).

### Isolation and identification of microbial strains

The identification of bacterial isolates from cucumber were performed using matrix assisted laser desorption ionization-time of flight-MS (MALDI–TOF–MS). About 10 g of cucumber stores were added to 90 mL sterile saline in 250 mL Erlenmeyer flask and incubated at 30 °C under shaking conditions (150 rpm) for 60 min. Serial dilution was performed and 0.1 mL was plated on nutrient agar plates. After 24 h growth, a series of purification was performed to obtain single separate colonies from each isolate. A single colony from each 24 h grown bacterial isolate culture plates was spotted on stainless-steel plate. α-Cyano-4-hydroxycinnamic acid (HCCA) matrix (1 µL) dissolved in 50% HPLC water, 47.5% ACN, 2.5% TFA and water was aliquoted on the colony and was left to dry at room temperature. The obtained Mass spectra were acquired using linear mode MALDI–TOF–MS (Scientific Analysis Instruments-LT3, United Kingdom) operating in linear mode and extracting positive ions with an accelerating voltage of 25 kV. The identification was performed and matched to BactoScreen database, scores above 0.8 are considered a match for both Genus and species.

### Extraction of anthocyanin from red cabbages

Fresh red cabbage (*Brassica oleracea* L.) was purchased from a local market in Cairo, Egypt. About 200 gm of freshly sliced red cabbage was mashed with 300 ml hot dist. water (80 °C) by an electric blender. The extract was filtered using Whatman paper no. 1, cooling at room temperature and centrifugation at 4000 rpm (4 °C for 10 min) then stored in clean dark bottles in the refrigerator (4 °C ± 1) for further study. The total anthocyanin (TAC) in red cabbage extract was calculated according to the following equation:

$$\Delta {\text{A}} = {\mkern 1mu} \left( {{\text{A}}_{{530}} - {\text{A}}_{{700}} } \right){\mkern 1mu} {\text{pH}}2 - \left( {{\text{A}}_{{530}} - {\text{A}}_{{700}} } \right){\mkern 1mu} {\text{pH}}6$$

$${\text{TAC }} = \frac{\Delta A \times MW \times DF \times 1000}{{\varepsilon \times L}}$$

where MW is the molecular weight of the pigment cyanidin-3-glucoside (449.2 g/mol), DF is the dilution factor, $\varepsilon$ is the extinction coefficient (26,900 L/cm mol) and L is the length of path (cm).

### Preparation of BC films

The BC film was prepared using a symbiotic culture of bacteria and yeast (SCOBY) according to a previous study (Hamed et al. 2023). BC nanofibrils produced in the form of gel-like pellicles (film) on the surface of sugared medium water (SW 6%) were collected and purified using (0.5 N) NaOH for removing the microbial cells and the residues of medium. Then, BC film was cut into small pieces (1.0 × 1.0 cm) and the patches were dried at 40–50 °C.

### Immobilization of red cabbage extract on BC films

Loading of AC (different adjusted pH) on BC patches was prepared by immersion of the dry BC patches in extract solution in a glass petri dish at room temperature overnight for maximum absorption of extract on the BC film. A part of the BC patches was allowed to air dry at 30 ± 2 °C and the second part was allowed to oven drying at 40–50 °C, while the last part was left in the wet condition without drying as a control. The obtained pH sensor was kept in a sealed container and dry place until use.

### Physiochemical analysis

At the beginning of analysis, the pH of all the samples was checked. Changes in pH value were determined using a Bench top pH meter (Wincom pH-8414 Portable pH meter) equipped with glassy pH electrode. Readings were taken at room temperature according to AOAC (2000). The pH meter was calibrated using a solution of known pH (4, 7 and 9).

### Gamma irradiation

Gamma irradiation was carried out using cobalt 60 irradiator source (Gamma Chamber 4000 India), located at National Center for Radiation Research and Technology (NCRRT), Nasr City, Cairo, Egypt, where the dose rate at the time of irradiation was 0.829 kGy/h. For each irradiated sample, one group was left without irradiation which was considered as control. The inoculated cucumber samples were exposed to gamma irradiation as sterilization dose (2 kGy), while RCE and RCE-BC were exposed to 2.5, 5. 10, 15, 20 and 25 kGy.

### Color change of red cabbage extract

The changes in red cabbage extract were monitored using UV–Visible spectrophotometer (SPECORD 210 plus, analytic Jena). Data plotted represents either change in extract spectrum within the visible region (400–800 nm) or reduction in color represented as decolorization. Decolorization was calculated according to the following equation:

$${\text{Decolorization}}\,\,\left( \% \right) = \frac{{{\text{Ai}} - {\text{Af}}}}{{{\text{AI}}}}$$

where A_i_ is the initial dye absorbance and A_f_ is the final dye absorbance.

### Statistical analysis

The significance of the data with different factors of non-irradiated and irradiated cucumbers samples was evaluated using Two-way analysis of variance ANOVA. All analysis was performed with SAS software package version 9 (SAS 2002).

## Results

### Isolation and identification of bacterial strain cucumbers and identification using MALDI–TOF

The microscopic examination of bacterial isolate from cucumber were conducted using standard Methods (Bartholomew and Mittwer 1952). The Identification of three bacterial isolates was confirmed by using MALDI-TOF MS data acquisition and processing. MALDI–TOF MS spectra were collected using a Microflex (SCI, UK) operating in linear positive ion detection mode located at National Center for Radiation Research and Technology (NCRRT), Nasr City, Cairo, Egypt. The count of each bacterial isolate for the upcoming experiments was 1.25 × 10^9^ for *Pseudomonas fluorescens,* 1.45 × 10^9^ for *Erwinia* sp and 1.40 × 10^9^ for *Pantoea agglomerans* (Table 1).

**Table 1:** MALDI–TOF identification of bacteria isolated from cucumber

No of isolates	Bacteria	Count (cfu/mL)	MALDI–TOF score
1	*Pseudomonas fluorescensw*	1.25 × 10^9^	0.82
2	*Erwinia* spp	1.45 × 10^9^	0.8
3	*Erwinia herbicola *(*Pantoea agglomerans*)	1.40 × 10^9^	0.9

### Effect of different pH values on red cabbage extract (RCE) color response in aqueous media and BC

**Fig. 1:**
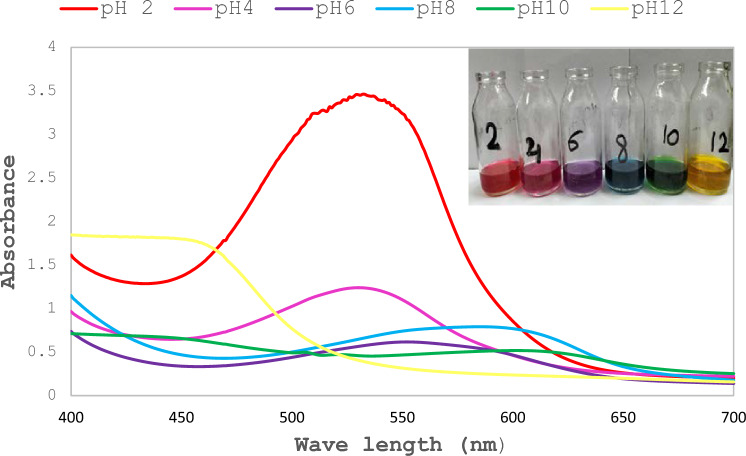
UV–Visible spectra of red cabbage extract at different pH values

**Fig. 2:**
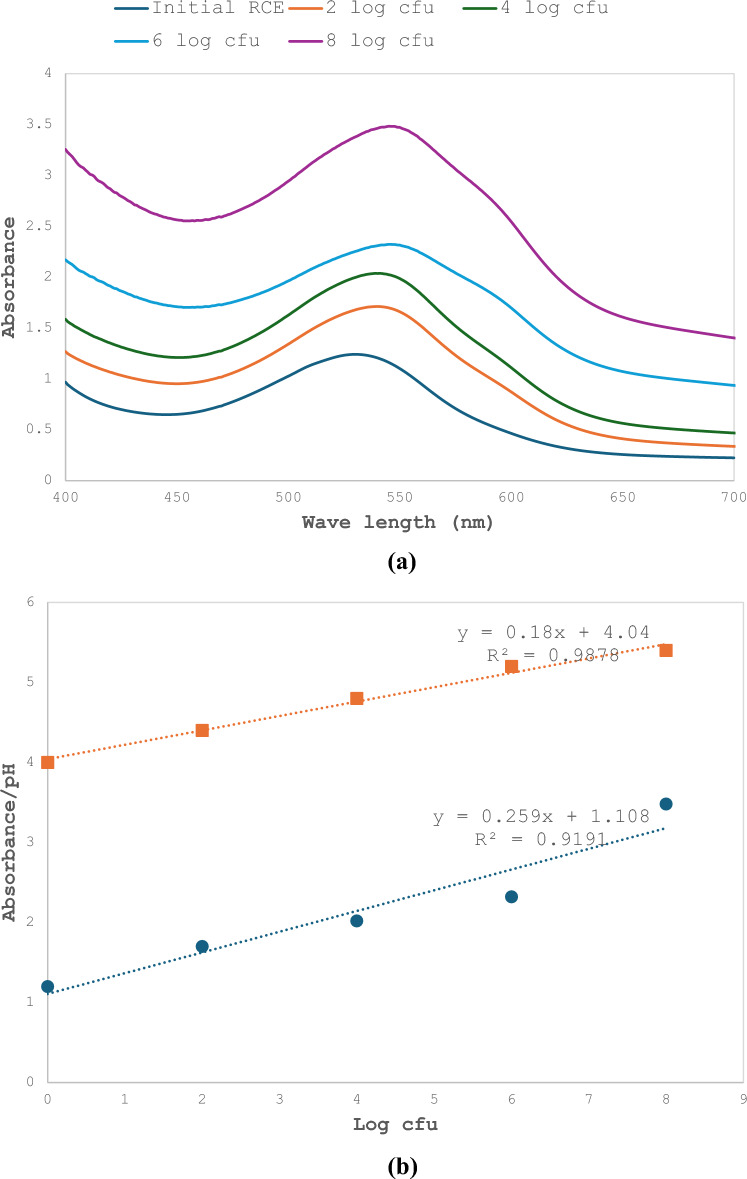
**a** UV–Visible spectra of serial dilutions of cucumber isolated bacterial mixture. **b** Correlation between maximum absorbance of red cabbage extract and serial dilutions of cucumber bacterial mixture calculated at 540 nm (blue filled circle) and pH values of each dilution (orange filled square)

The extracted total anthocyanin (TAC) pigment from red cabbage was calculated as 51.96 mg/L. This batch was used throughout the experiments. The spectra of the pH adjusted RCE across a range from 2 to 12 shows changes in absorbance maxima relevant to each color. pH values 2 and 4 were close and showed distinct peaks at 540 nm for red and pinkish red, respectively. RCE with pH value 6 showed a purple color with a distinct peak at 560 nm, pH 8 showed blue color with absorbance peak at 603 nm, pH 10 showed a green color with distinctive peak at 620 nm, while that for pH 12 showed a yellow color with distinctive peak at 463 nm (Fig. 1). Figure 2 represents immobilization of pH adjusted RCE on BC films. The visual results show that BC films acquired the same color as the aqueous solution. Since the isolated bacteria show pH values that ranged from 6 to 7 and preliminary tests showed an increase in acidity upon incubation, pH values 6 and above were excluded. pH 4 was chosen as the initial pH to detect visual changes in color for the upcoming experiments. It is noteworthy to mention that pH 2 was excluded as well, since it exhibits a close visual color as pH 4 but required more acid to obtain that value.

### Changes in RCE color in response to different bacterial count

The changes detected when the mixed bacterial culture was serially diluted were plotted and the results show that the absorption maxima increased with the increase in bacterial count. The recorded increase in absorbance peaks were 27.64, 38.5, 46.52 and 63.82% for log cfu/mL 2, 4, 6 and 8, respectively. It is notable that there was a slight shift in absorbance maxima towards the right which is another indication of the change in color (Fig. 2a). The correlation between the bacterial dilution values and pH values confirms that with serial dilutions of cucumber bacterial mixture, there was a concomitant decrease in pH was linear with R^2^ = 0.98 and that for bacterial count followed the same trend with R^2^ = 0.91 (Fig. 2b).

### RCE color changes of cucumbers at different storage time

**Fig. 3:**
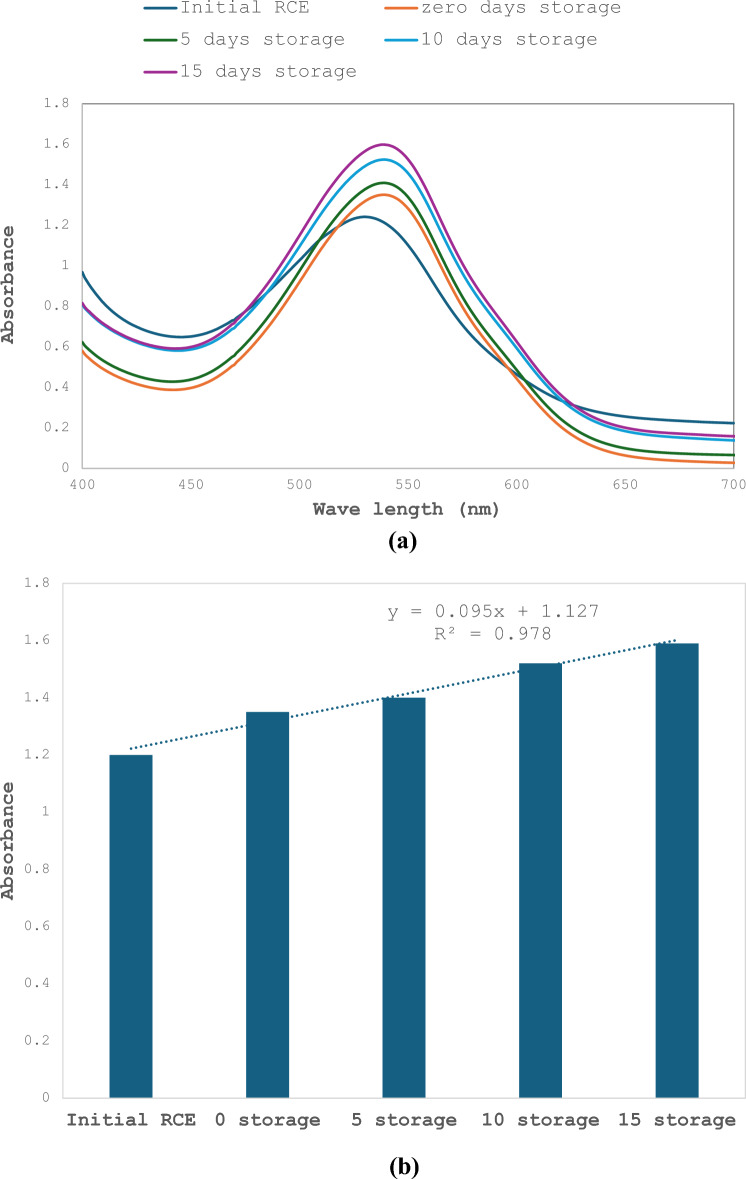
**a** UV visible spectra representing RCE absorbance changes of cucumbers at different. **b** Correlation between RCE absorbance and cucumber storage time in comparison with initial RCE

Aqueous RCE was tested to monitor changes in color when cucumbers were stored for 0, 5, 10 and 15 days. The results showed a slight increase in absorbance with the increase in storage time that reached 4, 10 and 15% after 5, 10 and 15 days. A slight shift to the right was notable, indicating change of color. The relationship between storage time and absorbance maxima showed R^2^ = 0.978 (Fig. 3a and b).

### RCE-BC color changes in response to different pHs

**Fig. 4:**
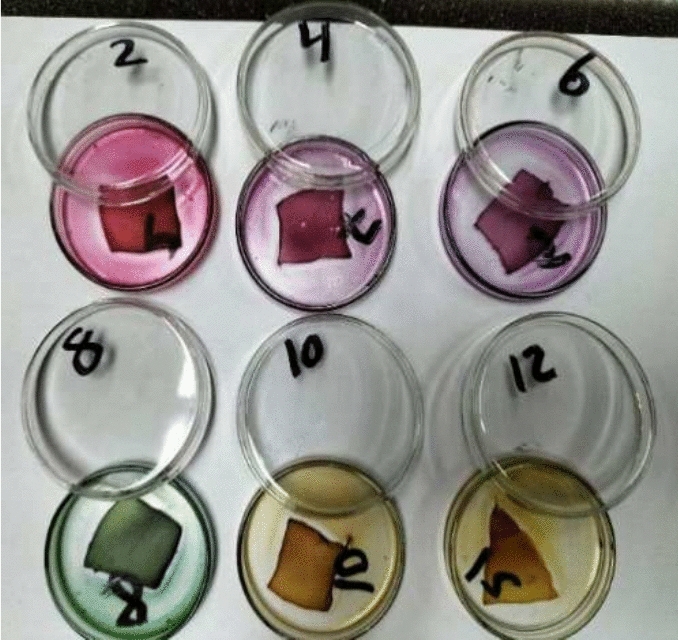
Immobilization of different pH adjusted red cabbage extract on bacterial cellulose films

BC prepared patches were immersed in different pH-adjusted RCE solutions. The color was detected visually as seen in Fig. 4.

### Effect of gamma radiation on RCE and RCE-BC

**Fig. 5:**
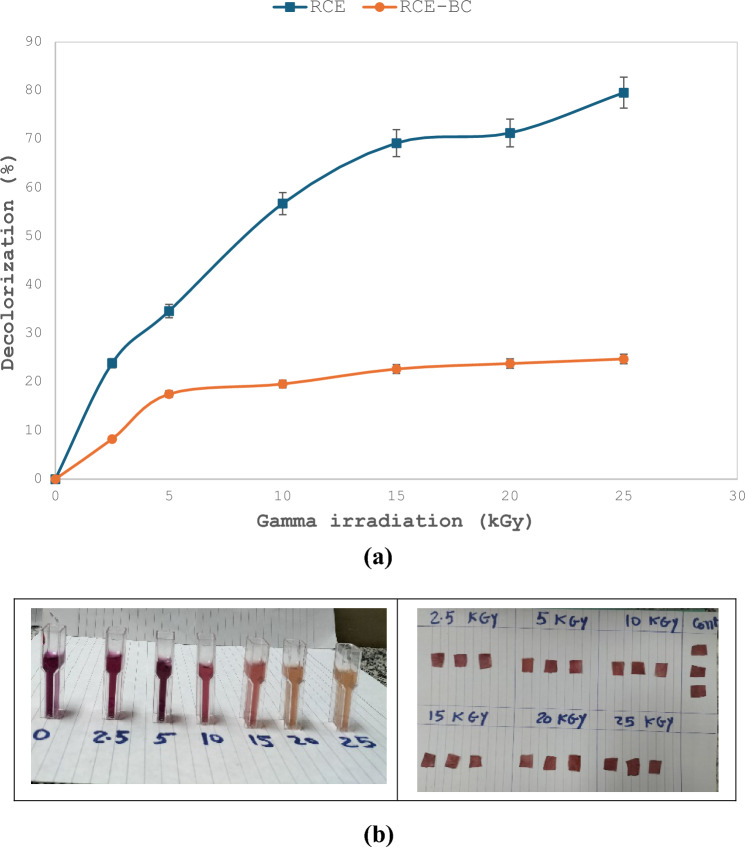
**a** Effect of gamma radiation on RCE and RCE-BC color changes. **b** Color response of RCE and RCE-BC after exposure to different gamma radiation doses

Figure 5a shows that exposure to increasing gamma radiation doses decreased the color of both RCE and RCE-BC, however, the response of RCE to gamma irradiation was threefold higher than that for RCE-BC. Images shown in Fig. 5b represent the color changes.

### Effect of storage time with and without gamma irradiation of cucumbers on RCE-BC

**Fig. 6:**
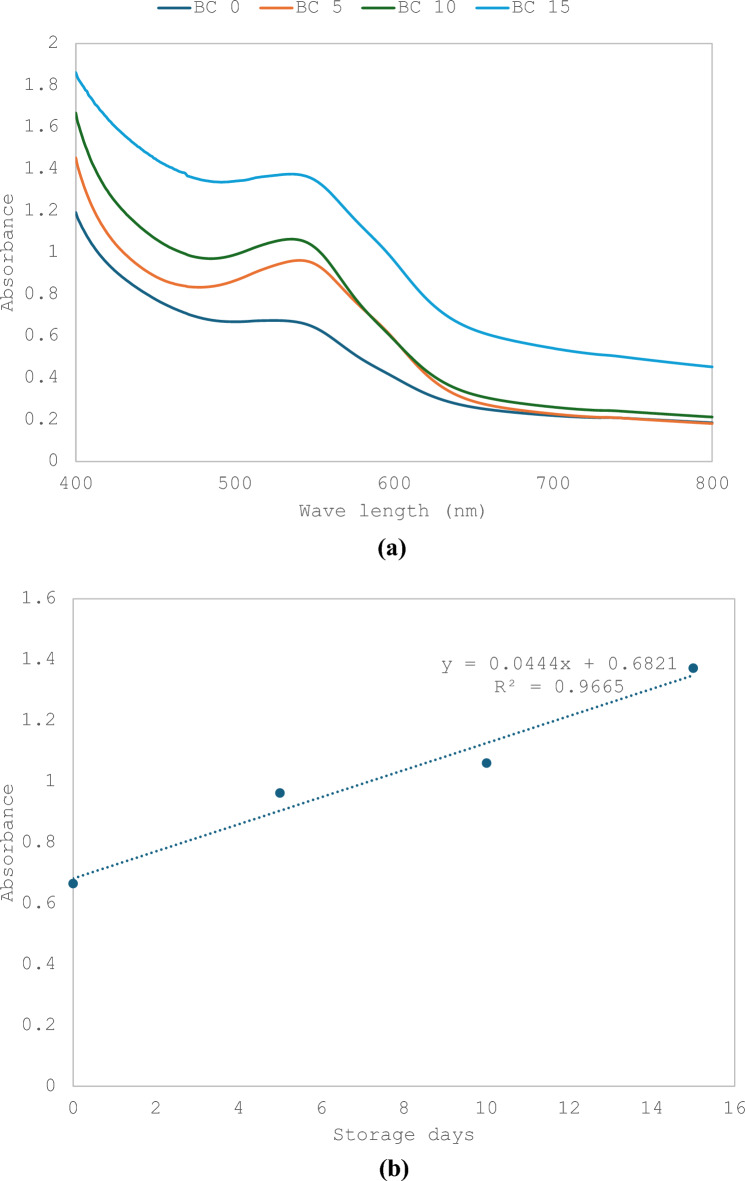
**a** UV–Visible spectra representing changes of bacterial cellulose immobilized red. **b** Correlation between bacterial cellulose immobilized red extract absorbance and storage days of cucumbers calculated at 540 nm

**Fig. 7:**
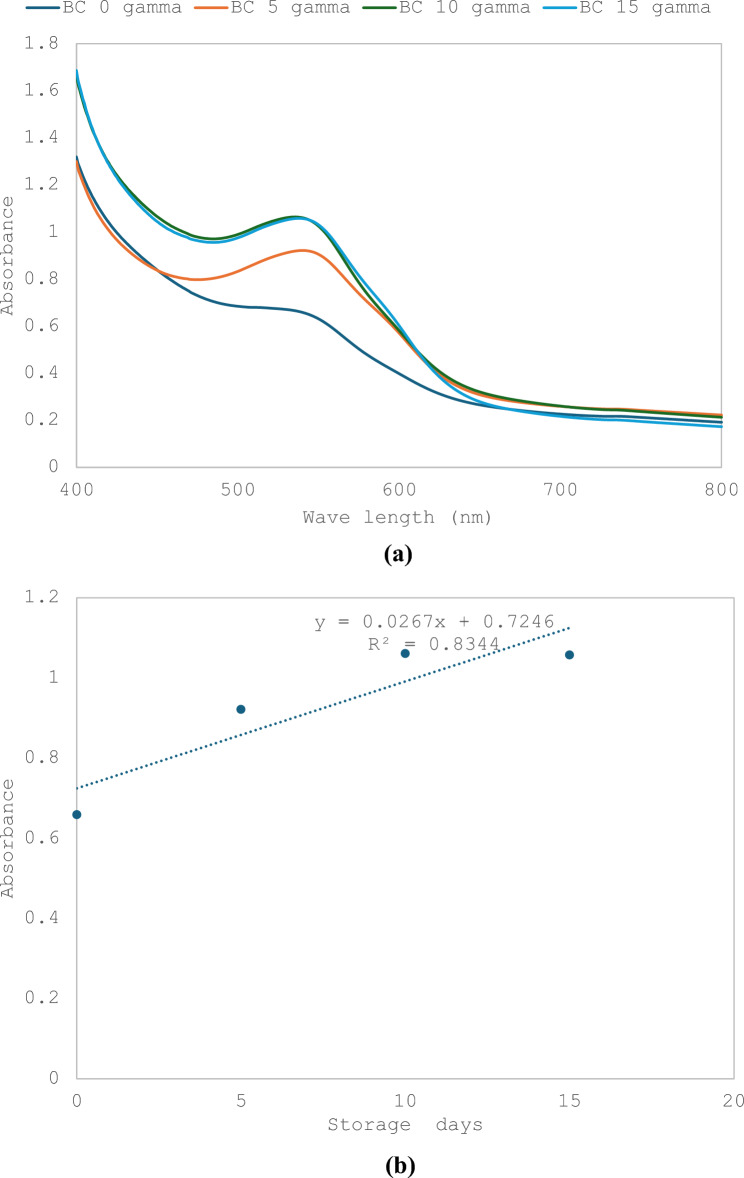
**a** UV–Visible spectra representing changes of bacterial cellulose immobilized red. **b** Correlation between bacterial cellulose immobilized red extract absorbance and storage days of 2 kGy irradiated cucumbers calculated at 540 nm

RCE-BC placed on cucumbers were stored for 0, 5, 10 and 15 days. The results showed an increase in absorbance with the increase in storage time that reached 37.5, 42 and 56% after 5, 10 and 15 days. The relationship between storage time and absorbance maxima showed R^2^ = 0.96. (Fig. 6a and b). On the other hand, when 2 kGy gamma irradiated cucumbers were mixed with aqueous RCE, the results showed a slight decrease in absorbance with the increase in storage time that reached 30 after storage for 5 days and 40% for both 10 and 15 days. The relationship between storage time and absorbance maxima showed R^2^ = 0.834 (Fig. 7a and b). The results in Table 2 show that the total aerobic mesophilic bacteria count was reduced by almost 4 log cycles upon 2 kGy gamma irradiation for each storage period. However, the overall log count increased for both non-irradiated and 2 kGy gamma irradiated RCE-BC with the increase in storage time. Table 2 represents the increase in bacterial count in both cases. The results show that there is a fourfold increase in bacterial count when comparing bacterial count at each storage time with and without gamma irradiation. On the other hand, there was a gradual increase in bacterial count with storage time for both groups.

**Table 2:** Effect of γ-irradiation (2 kGy) and subsequent storage (4 °C ± 1) on the microbial counts (log cfu/mL) mixture contaminating cucumber

Micro-organisms	Storage (days)	Irradiation dose (kGy)	
		0.0	2.0
Total aerobic mesophilic bacteria mixture	0	6.20^aa^ ± 0.06	2.17^ba^ ± 0.01
	5	7.07^ab^ ± 0.02	3.34^bb^ ± 0.04
	10	8.20^ac^ ± 0.09	4.51^bc^ ± 0.03
	15	9.13^ad^ ± 0.01	5.47^bd^ ± 0.07

## Discussion

The use of natural dyes as visual indicators remains as one of the innovative approaches in food packaging methods. Commercial packaging with pH sensors depends on the sensitive nature of dyes and their change according to pH (Oladzadabbasabadi et al. 2022). These pH sensors are very important to detect spoilage early in post-harvest produce such as cucumbers that are not usually visible during early storage, therefore, the first aim of the present study is to use pH sensitive indicator to detect microbial contamination of refrigerator stored cucumbers and assess cucumber freshness visually. Microbial load depends on the irrigation water used and whether its clean water or wastewater treated (Gurtler and Gibson 2022). This means that the irrigation water will determine the microorganisms present and that they will vary each time. In the present study, the microbial load of the cucumbers in the present study was identified three main microorganisms; *Pseudomonas fluorescens, Erwinia* sp. and *Pantoea agglomerans* as predominant strains based on MALDI–TOF–MS data acquisition and processing spectra. MALDI–TOF–MS is a powerful tool for rapid bacterial identification (Ashfaq et al. 2022). This method is not only rapid and cost efficient but is more appropriate to detect foodborne pathogens in the food chain complex as well (Horváth et al. 2020). Microbes generally interact with each other and with their surrounding media utilizing nutrients and producing metabolites modifying their surrounding media. The most common media transformation is pH change of the media that is influenced by the present metabolites produced during microbial growth (Ratzke and Gore 2018). Since the aim of our work is to detect microbial contamination based on the pH changes, it was crucial to choose RCE adjusted at pH that would give a different color than that of the final pH of the cucumber microbial load. Therefore, pH 4 was chosen for RCE since the final pH value of the three identified microbes ranged from 6 to 7. RCE contains anthocyanin pigment which shows unique color changes over a wide pH range. The extract is red at low pH, changing to blue at intermediate pH, and turns green at higher pH values (Khoo et al. 2017). This color change is attributed to the structure of anthocyanins and their hyperchromic and bathochromic properties (Ghareaghajlou et al. 2021). Anthocyanin is one of the bioactive compounds that can be used to monitor food freshness since it is pH sensitive and at the same time safe to use (Wulandari et al. 2020). The results obtained show different color spectra after adjusting the pH values of RCE. Anthocyanins naturally occur as anthocyanidins, its basic structure is 2-phenyl-benzopyrylium chromophore–flavylium ion. Variation of pH changes the molecular structure of anthocyanin resulting in different colors. This pH responsive nature prompted great attention to its use in smart food packaging as an indicator to food spoilage. The main mechanism depends on the increase of volatile amines produced by bacteria which result in increase in pH and therefore induce a color change (Oladzadabbasabadi et al. 2022). Anthocyanins naturally form stable red color (flavylium cation) at pH < 4. Their color changes to purple/blue quinoidal anhydro base at pH 6 to 8. A yellow and light-yellow or colorless chalcone can be visually seen at pHs above 8 (Rawdkuen et al. 2020). However, the pallet color of anthocyanin at different pH values changes with the change of the anthocyanin source of extraction (Oladzadabbasabadi et al. 2022), therefore, it was essential to do this experiment for both RCE and RCE incorporated within BC to identify the changes that will take place for cucumbers.

RCE pigment incorporation within bacterial cellulose (BC) did not affect the anthocyanin response to pH adjustments. This reveals that anthocyanin activity inside the BC structure was protected and not affected by the interaction with BC matrix. A study conducted by Divsalar et al. (2017) and Lotfi et al. (2018) revealed no change in FTIR spectra for anthocyanin pigment incorporated within BC film. There were no reported changes to BC structure as well (Kuswandi et al. 2020). The 3D porous structure of BC nanofibers (120–160 nm) allows the diffusion of anthocyanin pigment within the inner network space. These features may increase the absorption of liquids that are caused by pH change and accelerate the transformation of the color of the dye immobilized in the BC film resulting in the development of highly sensitive and rapid response pH sensor. Both RCE and RCE-BC films exhibited visual changes in color with the increase in microbial contamination changed from deep pink to purple, further increase to alkaline pH changes the color to green. Color change in RCE and RCE-BC was evident as early as 5-day incubation. This makes it very useful to detect contamination at early stages since cucumbers exhibit visual morphological changes after 10 and 15 days of incubation. Therefore, RCE and RCE-BC films can be used to detect microbial contamination of stored cucumbers.

Food irradiation is considered one of the effective and accredited methods that ensures food quality and food safety as well as increases shelf-life (Khalili et al. 2017). The second aim of the present work was to investigate if RCE and RCE-BC can be utilized to detect if gamma irradiation was used for cucumber preservation. The results obtained show that exposure of cucumbers to 2 kGy gamma irradiation treatment reduced the total mesophilic bacteria mixture by almost 4 log cycle as compared to non-irradiated cucumbers, this resulted in prolonging the shelf life of cucumber to 15 days. A preservation dose of 2 kGy was reported to be the optimal irradiation dose that decontaminates cucumbers with less impact on tissue firmness and flavor (Khalili et al. 2017). Ionizing radiation can change physical, chemical, and biological characteristics of materials controlled by the absorbed doses, therefore, it could be a good indicator and can be used for different radiation dosimetry applications (Aljoumaa and Khalil 2019). The present study shows a correlation between RCE maximum absorbance and different gamma irradiation doses where the increase in gamma radiation doses resulted in the decrease of RCE color. This decrease can be attributed to the nature of anthocyanin and its polyphenolic structures which are sensitive to oxidation during irradiation (Hooshyar et al. 2020). On the other hand, RCE-BC showed less color changes when compared to RCE in its aqueous form at low radiation concentrations, but somehow visible changes tool place at higher radiation doses. This suggests that there is a need for more research to develop RCE-BC films that exhibit higher color changes when exposed to low preservation doses.

Anthocyanin-polymer film is affected by different factors such as the source of anthocyanin, extraction procedure (Prietto et al. 2017), the interaction with the polymer functional groups, co-film-forming agents (e.g., plasticizers, nanoparticles, crosslinkers, etc.), and the present water molecules (Chen et al. 2021). The bonds forming between the aromatic rings of anthocyanin and the glucose units in BC film result from the polar interactions between anions of negatively charged hydroxyl groups (OH) present in BC structures and cations of positively charged oxygen in central pyran ring in anthocyanin (Pismensykaia et al. 2020). The hydroxyl groups in anthocyanin and the functional groups in polymeric matrix were found to be associated physically by inter- and intra-molecular connections, such as electrostatic interactions and hydrogen bonds which are weaker than the covalent bonds and often cause different changes in the physical, mechanical, thermal properties and structure of the polymer (Abedi-Firoozjah et al. 2022). It was reported that the incorporation of anthocyanin (AC) into bacterial cellulose (BC) film may reduce the BC macromolecules interactions due to the presence of phenolic compounds which act as plasticizers (Kuswandi et al. 2020; Abedi-Firoozjah et al. 2022). Color stability is an essential factor in establishing colorimetric pH sensors that can be used in intelligent food packaging systems and show color stability during storage (Ezati et al. 2019). The chemical structure and properties of cellulose protects RCE from degradation. Zhao et al. (2019) stated that cellulosic coating solutions make a powerful barrier after drying. This barrier protects the pigment and decreases its leaching during thermal or any other processing of food stuff.

The idea of incorporating natural anthocyanin pigment within a biopolymer has been previously reported yet BC use as matrix for RCE is still considered advantageous. BC production is a natural process and simple to obtain compared to other matrixes that involve the multi-steps for preparation (Wu et al. 2022) or extensive extraction and preparation process (Khezerlou et al. 2023). BC is produced naturally using a mixture of symbiotic colony of bacteria and yeast (SCOBY) using sugared water as simple production media. It forms sheets on the surface of the media (Hamed et al. 2023) which make it easy to collect and use as matrix for incorporating RCE. Therefore, the present study proves that RCE anthocyanin liquid or RCE incorporated within BC are efficient, cost-effective, edible and ecofriendly compared to other pH sensors reported in other studies. This is the first report for anthocyanin incorporated in BC that can be used for 1) detection of cucumber spoilage in refrigerated produce and 2) identifying if the cucumbers were exposed to gamma radiation.

## Conclusion

According to the United Nations Sustainable Development Goal 12: responsible consumption and production, target 12.3 aims at halving global food waste by 2030 at retail and consumer level. The main objective is to reduce food loss throughout the supply chain. The current work represents a novel pH-sensitive indicator that senses both contamination and gamma radiation. The biomaterial used was SCOBY produced BC which makes the sensor eco-friendly. The color change of RCE is visible and. offers a time-resolving indicator for both cucumber freshness and gamma irradiation. The relationship between pH changes and bacterial count was directly proportional. The same applies to color change after 2-week stored non-irradiated and gamma irradiated RCE-BC films on cucumbers. The prepared RCE-BC can be used as a label or further developed to coat produce and can be used as intelligent packaging to indicate freshness and avoid food loss. RCE was successful in detecting gamma radiation exposure at both low and high doses. However, there is a need for further development of RCE-BC to produce films that can detect low dose gamma irradiation by the naked eye. Further development for the prepared RCE-BC involves its antioxidant activity and response to different storage conditions such as temperature variation, humidity, light, and stability under different storage conditions. This will be the focus of our upcoming work.

## Data Availability

The data obtained will be available upon request.

## Abbreviations

RCE: Red cabbage extract
BC: Bacterial cellulose
RCE-BC: Red cabbage extract incorporated within BC films
kGy: Kilo Gray
TPCs: Total plate counts
MALDITOF MS: Matrix-assisted laser desorption ionization–time of flight-mass spectrometry
ACN: Acetonitrile
TFA: Trifluoroacetic acid
HCCA: α-Cyano-4-hydroxycinnamic acid
HPLC: High performance liquid chromatography
FLW: Food loss and waste
IP: Intelligent packaging
FAO: Food and Agriculture Organization
AC: Anthocyanin
TAC: Total anthocyanin
MW: Molecular weight
DF: Dilution factor
ε: Extinction coefficient
L: Length of path
SCOBY: Symbiotic culture of bacteria and yeast
SW: Sugared water
AOAC: Association of Official Analytical Chemists
CFU: Colony forming unit
3D: Three dimensional
PVA: Polyvinyl alcohol
RCP: Red cabbage puree

## References

[CR1] Abedi-Firoozjah R, Yousefi S, Heydari M, Seyedfatehi F, Jafarzadeh S, Mohammadi R, Garavand F (2022) Application of red cabbage anthocyanins as pH-sensitive pigments in smart food packaging and sensors. Polymers 14(8):1629. 10.3390/polym14081629 35458378 10.3390/polym14081629 PMC9025686

[CR2] Aljoumaa K, Khalil A (2019) Evaluations of red cabbage (*Brassica oleracea* L. Var. Capitata F. Rubra DC.) extract behavior under high dose gamma irradiation. Int Res j Pure Appl Chem 17(4):1–9. 10.9734/IRJPAC/2018/46199 10.9734/IRJPAC/2018/46199

[CR3] AOAC (2000) Official methods of analysis, 16th edn. Association of Official Analytical Chemists, Rockville

[CR4] APHA (1992) Standard methods for the examination of dairy products. American Public Health Association, Washington, D.C.

[CR5] Ashfaq MY, Da’na DA, Al-Ghouti MA (2022) Application of MALDI–TOF MS for identification of environmental bacteria: a review. J Environ Manage 1(305):114359. 10.1016/j.jenvman.2021.114359 10.1016/j.jenvman.2021.114359 34959061

[CR6] Bartholomew JW, Mittwer T (1952) The gram stain. Bact Rev 16:1 14925025 10.1128/br.16.1.1-29.1952 PMC180726

[CR7] Beretta C, Stucki M, Hellweg S (2017) Environmental impacts and hotspots of food losses: value chain analysis of Swiss food consumption. Environ Sci Technol 51(19):11165–11173 28862841 10.1021/acs.est.6b06179

[CR8] Chen HZ, Zhang M, Bhandari B, Guo Z (2018) Applicability of a colorimetric indicator label for monitoring freshness of fresh-cut green bell pepper. Postharvest Biol Technol 140:85–92 10.1016/j.postharvbio.2018.02.011

[CR9] Chen M, Yan T, Huang J, Zhou Y, Hu Y (2021) Fabrication of halochromic smart films by immobilizing red cabbage anthocyanins into chitosan/oxidized-chitin nanocrystals composites for real-time hairtail and shrimp freshness monitoring. Int J Biol Macromol 179:90–100. 10.1016/j.ijbiomac.2021.02.170 33636274 10.1016/j.ijbiomac.2021.02.170

[CR10] Choi SM, Rao KM, Zo SM, Shin EJ, Han SS (2022) Bacterial cellulose and its applications. Polymers 14(6):1080. 10.3390/polym14061080 35335411 10.3390/polym14061080 PMC8949969

[CR11] Dirpan A, Latief R, Syarifuddin A, Rahman AN, Putra RP, Hidayat SH (2018) The use of colour indicator as a smart packaging system for evaluating mangoes Arummanis (*Mangifera indica L var Arummanisa*) freshness. IOP Conf Ser Earth Environ Sci 1(157):012031. 10.1088/1755-1315/157/1/012031 10.1088/1755-1315/157/1/012031

[CR12] Divsalar E, Tajik H, Moradi M, Forough M, Lotfi M, Kuswandi B (2017) Characterization of cellulosic paper coated with chitosan-zinc oxide nanocomposite containing nisin and its application in packaging of UF cheese. Int J Biol Macromol 109:1311–1318 29175522 10.1016/j.ijbiomac.2017.11.145

[CR13] Dos Santos SF, Cardoso RDCV, Borges ÍMP, e Almeida AC, Andrade ES, Ferreira IO, do Carmo RL (2020) Post-harvest losses of fruits and vegetables in supply centers in Salvador, Brazil: analysis of determinants, volumes and reduction strategies. Waste Manage 101:161–170. 10.1016/j.wasman.2019.10.007 10.1016/j.wasman.2019.10.007 31610477

[CR14] El-Dkeshy MH, Haridy AG, Abbas HS, Abdel-Hakim KA (2023) Impact of seedling dates and different foliar applications on growth and productivity of cucumber hybrids (Barracuda.) under protected agricultural systems. Assiut J Agri Sci. 10.21608/AJAS.2023.206597.1247 10.21608/AJAS.2023.206597.1247

[CR15] Ezati P, Tajik H, Moradi M, Molaei R (2019) Intelligent pH-sensitive indicator based on starch-cellulose and alizarin dye to track freshness of rainbow trout fillet. Int J Biol Macromol 1(132):157–165. 10.1016/j.ijbiomac.2019.03.173 10.1016/j.ijbiomac.2019.03.173 30926497

[CR61] FAO Statistics, Food and Agriculture Organization of the United Nations, Rome. https://www.fao.org/faostat/en/#data/QCL

[CR16] Gasti T, Dixit S, D’souza OJ, Hiremani VD, Vootla SK, Masti SP, Chougale RB, Malabadi RB (2021) Smart biodegradable films based on chitosan/ methylcellulose containing *Phyllanthus reticulatus* anthocyanin for monitoring the freshness of fish fillet. Int J Biol Macromol 187:451–461. 10.1016/j.ijbiomac.2021.07.128 34324903 10.1016/j.ijbiomac.2021.07.128

[CR17] Ghareaghajlou N, Hallaj-Nezhadi S, Ghasempour Z (2021) Red cabbage anthocyanins: stability, extraction, biological activities and applications in food systems. Food Chem 365:130482. 10.1016/j.foodchem.2021.130482 34243124 10.1016/j.foodchem.2021.130482

[CR18] Gurtler JB, Gibson KE (2022) Irrigation water and contamination of fresh produce with bacterial foodborne pathogens. Curr Opin Food Sci. 10.1016/j.cofs.2022.100889 10.1016/j.cofs.2022.100889

[CR19] Halász K, Kóczán Z, Joóbné Preklet E (2023) pH-dependent color response of cellulose-based time-temperature indicators impregnated with red cabbage extract. Food Meas 17:2555–2565. 10.1007/s11694-023-01805-y 10.1007/s11694-023-01805-y

[CR20] Hamed DA, Maghrawy HH, Abdel Kareem H (2023) Biosynthesis of bacterial cellulose nanofibrils in black tea media by a symbiotic culture of bacteria and yeast isolated from commercial kombucha beverage. World J Microbiol Biotechnol 39(2):48. 10.1007/s11274-022-03485-0 10.1007/s11274-022-03485-0 PMC9768004 36538179

[CR21] Hooshyar L, Hesari J, Azadmard-Damirchi S (2020) Investigation of selected thermal and non-thermal preservative techniques to produce high quality and safe to drink sour cherry, red grape and pomegranate juices. J Food Sci Technol 57:1689–1697. 10.1007/s13197-019-04202-w 32327780 10.1007/s13197-019-04202-w PMC7171010

[CR22] Horváth B, Peles F, Szél A, Sipos R, Erős Á, Albert E, Micsinai A (2020) Molecular typing of foodborne coagulase-positive *Staphylococcus* isolates identified by MALDI–TOF MS. Acta Aliment. 10.1556/066.2020.49.3.9 10.1556/066.2020.49.3.9

[CR23] Ito VC, Zielinski AAF, Demiate IM, Spoto M, Nogueira A, Lacerda LG (2019) Effects of gamma radiation on the stability and degradation kinetics of phenolic compounds and antioxidant activity during storage of (*Oryza sativa *L) black rice flour. Braz Arch Biol Technol. 10.1590/1678-4324-2019180470 10.1590/1678-4324-2019180470

[CR24] Jang EJ, Padhan B, Patel M, Pandey JK, Xu B, Patel R (2023) Antibacterial and biodegradable food packaging film from bacterial cellulose. Food Control. 10.1016/j.foodcont.2023.109902 10.1016/j.foodcont.2023.109902

[CR25] Khalili R, Ayoobian N, Jafarpour M, Shirani B (2017) The effect of gamma irradiation on the properties of cucumber. J Food Sci Technol 54(13):4277–4283. 10.1007/s13197-017-2899-7 29184234 10.1007/s13197-017-2899-7 PMC5686008

[CR26] Khezerlou A, Tavassoli M, Alizadeh Sani M, Ehsani A, McClements DJ (2023) Smart packaging for food spoilage assessment based on *Hibiscus sabdariffa* L anthocyanin-loaded chitosan films. J Compos Sci 7(10):404. 10.3390/jcs7100404 10.3390/jcs7100404

[CR27] Khoo HE, Azlan A, Tang ST, Lim SM (2017) Anthocyanidins and anthocyanins: colored pigments as food, pharmaceutical ingredients, and the potential health benefits. Food Nutr Res 61(1):1361779. 10.1080/16546628.2017.1361779 28970777 10.1080/16546628.2017.1361779 PMC5613902

[CR28] Kossyvaki D, Contardi M, Athanassiou A, Fragouli D (2022) Colorimetric indicators based on anthocyanin polymer composites a review. Polymers 14(19):4129. 10.3390/polym14194129 36236076 10.3390/polym14194129 PMC9571802

[CR29] Kusumowardani N, Tjahjono B, Lazell J, Bek D, Theodorakopoulos N, Andrikopoulos P, Priadi CR (2022) A circular capability framework to address food waste and losses in the agri-food supply chain: the antecedents, principles and outcomes of circular economy. J Bus Res 142:17–31. 10.1016/j.jbusres.2021.12.020 10.1016/j.jbusres.2021.12.020

[CR30] Kuswandi B, Asih NP, Pratoko DK, Kristiningrum N, Moradi M (2020) Edible pH sensor based on immobilized red cabbage anthocyanins into bacterial cellulose film for intelligent food packaging. Packag Technol Sci 33:321–332. 10.1002/pts.2507 10.1002/pts.2507

[CR31] Lee K, Park H, Baek S, Han S, Kim D, Chung S, Yoon JY, Seo J (2019) Colorimetric array freshness indicator and digital color processing for monitoring the freshness of packaged chicken breast. Food Packag Shelf Life 22:100408. 10.1016/j.fpsl.2019.100408 10.1016/j.fpsl.2019.100408

[CR32] Lotfi M, Tajik H, Moradi M, Forough M, Divsalar E, Kuswandi B (2018) Nanostructured chitosan/monolaurin film: preparation, characterization and antimicrobial activity against listeria monocytogenes on ultrafiltered white cheese. LWT 92:576–583. 10.1016/j.lwt.2018.03.020 10.1016/j.lwt.2018.03.020

[CR33] Lu P, Yang Y, Liu R, Liu X, Ma J, Wu M, Wang S (2020) Preparation of sugarcane bagasse nanocellulose hydrogel as a colourimetric freshness indicator for intelligent food packaging. Carbohydr Polym 249:116831. 10.1016/j.carbpol.2020 32933676 10.1016/j.carbpol.2020

[CR34] Maftoonazad N, Ramaswamy H (2019) Design and testing of an electrospun nanofiber mat as a pH biosensor and monitor the pH associated quality in fresh date fruit (Rutab). Polym Test 1(75):76–84. 10.1016/j.polymertesting.2019.01.011 10.1016/j.polymertesting.2019.01.011

[CR35] Merz B, Capello C, Leandro GC, Moritz DE, Monteiro AR, Valencia GA (2020) A novel colorimetric indicator film based on chitosan, polyvinyl alcohol and anthocyanins from jambolan (*Syzygium cumini*) fruit for monitoring shrimp freshness. Int J Biol Macromol 153:625–632. 10.1016/j.ijbiomac.2020.03.048 32165201 10.1016/j.ijbiomac.2020.03.048

[CR36] Mohamed SA, El-Sakhawy M, El-Sakhawy MA (2020) Polysaccharides, protein and lipid-based natural edible films in food packaging: a review. Carbohydr Polym 238:116178. 10.1016/j.carbpol.2020.116178 32299560 10.1016/j.carbpol.2020.116178

[CR37] Musso YS, Salgado PR, Mauri AN (2017) Smart edible films based on gelatin and curcumin. Food Hydrocoll 66:8–15. 10.1016/j.foodhyd.2016.11.007 10.1016/j.foodhyd.2016.11.007

[CR38] Oladzadabbasabadi N, Nafchi AM, Ghasemlou M, Ariffin F, Singh Z, Al-Hassan AA (2022) Natural anthocyanins: sources, extraction, characterization, and suitability for smart packaging. Food Packag Shelf Life 33:100872 10.1016/j.fpsl.2022.100872

[CR39] Pirsa S, Sani IK, Pirouzifard MK, Erfani A (2020) Smart film based on chitosan/melissa officinalis essences/pomegranate peel extract to detect cream cheeses spoilage, food addit. Contam 37:634–648. 10.1080/19440049.2020.1716079 10.1080/19440049.2020.1716079 31971478

[CR40] Pismenskaya N, Sarapulova V, Klevtsova A, Mikhaylin S, Bazinet L (2020) Adsorption of anthocyanins by cation and anion exchange resins with aromatic and aliphatic polymer matrices. Int J Mol Sci 21(21):7874. 10.3390/ijms21217874 33114195 10.3390/ijms21217874 PMC7660631

[CR41] Pourjavaher S, Almasi H, Meshkini S, Pirsa S, Parandi E (2017) Development of a colorimetric pH indicator based on bacterial cellulose nanofibers and red cabbage (*Brassica oleraceae*) extract. Carbohydr Polym 156:193–201. 10.1016/j.carbpol.2016.09.027 27842814 10.1016/j.carbpol.2016.09.027

[CR42] Prietto L, Mirapalhete TC, Pinto VZ, Hoffmann JF, Vanier NL, Lim LT, Dias ARG, da Rosa ZE (2017) pH-sensitive films containing anthocyanins extracted from black bean seed coat and red cabbage. LWT 80:492–500. 10.1016/J.LWT.2017.03.006 10.1016/J.LWT.2017.03.006

[CR43] Priyadarshi R, Ezati P, Rhim JW (2021) Recent advances in intelligent food packaging applications sing natural food colorants. ACS Food Sci Technol 1(2):124–138. 10.1021/acsfoodscitech.0c00039 10.1021/acsfoodscitech.0c00039

[CR44] Ratzke C, Gore J (2018) Modifying and reacting to the environmental pH can drive bacterial interactions. PLoS Biol 16(3):2004248. 10.1371/journal.pbio.2004248 10.1371/journal.pbio.2004248 PMC5868856 29538378

[CR45] Rawdkuen S, Faseha A, Benjakul S, Kaewprachu P (2020) Application of anthocyanin as a color indicator in gelatin films. Food Biosci. 36:100603 10.1016/j.fbio.2020.100603

[CR46] Rukchon C, Nopwinyuwong A, Trevanich S, Jinkarn T, Suppakul P (2014) Development of a food spoilage indicator for monitoring freshness of skinless chicken breast. Talanta 130:547–554. 10.1016/j.talanta.2014.07.048 25159445 10.1016/j.talanta.2014.07.048

[CR62] SAS (Statistical Analysis Software) (2002) Statistical Analysis Software Guide for Personal Computers. Release 9.1, SAS Institute Inc., Cary

[CR47] Silva-Pereira MC, Teixeira JA, Pereira- Júnior VA, Stefani R (2015) Chitosan/corn starch blend films with extract from *Brassica oleraceae* (red cabbage) as a visual indicator of fish deterioration. LTW-Food Sci Tech 61:258–262. 10.1016/j.lwt.2014.11.041 10.1016/j.lwt.2014.11.041

[CR48] Sobhan A, Muthukumarappan K, Wei L (2021) Biosensors and biopolymer-based nanocomposites for smart food packaging: challenges and opportunities. Food Packag Shelf Life 30:100745 10.1016/j.fpsl.2021.100745

[CR49] Sobhan A, Muthukumarappan K, Wei L (2022) A biopolymer-based pH indicator film for visually monitoring beef and fish spoilage. Food Biosci 46:101523. 10.1016/j.fbio.2021.101523 10.1016/j.fbio.2021.101523

[CR50] Taherkhani E, Moradi M, Tajik H, Molaei R, Ezati P (2020) Preparation of on package halochromic freshness/spoilage nanocellulose label for the visual shelf life estimation of meat. Int J Biol Macromol 164:2632–2640. 10.1016/j.ijbiomac.2020.08.177 32853605 10.1016/j.ijbiomac.2020.08.177

[CR51] Villarreal-soto SA, Beaufort S, Bouajila J, Souchard J, Renard T, Rollan S, Taillandier P (2019) Impact of fermentation conditions on the production of bioactive 662 compounds with anticancer, anti-inflammatory and antioxidant properties in kombucha 663 tea extracts. Process Biochem. 10.1016/j.procbio.2019.05.004 10.1016/j.procbio.2019.05.004

[CR52] Wang L, Yang C, Deng X, Peng J, Zhou J, Xia G, Yang H (2023) A pH-sensitive intelligent packaging film harnessing *Dioscorea zingiberensis* starch and anthocyanin for meat freshness monitoring. Int J Biol Macromol. 10.1016/j.ijbiomac.2023.125485 37348585 10.1016/j.ijbiomac.2023.125485

[CR53] Weston M, Phan MAT, Arcot J, Chandrawati R (2020) Anthocyanin-based sensors derived from food waste as an active use-by date indicator for milk. Food Chem 326:127017 32434111 10.1016/j.foodchem.2020.127017

[CR54] Wu H, Jiao C, Li S, Li Q, Zhang Z, Zhou M, Yuan X (2022) A facile strategy for development of pH-sensing indicator films based on red cabbage puree and polyvinyl alcohol for monitoring fish freshness. Foods 11(21):3371. 10.3390/foods11213371 36359984 10.3390/foods11213371 PMC9653917

[CR55] Wulandari A, Sunarti TC, Fahma F, Enomae T (2020) The potential of bioactives as biosensors for detection of pH. In IOP Conf Ser Earth Environ 460(1):012034. 10.1088/1755-1315/460/1/012034 10.1088/1755-1315/460/1/012034

[CR56] Yousefi H, Ali MM, Su HM, Filipe CDM, Didar TF (2018) Sentinel Wraps: real-time monitoring of food contamination by printing DNAzyme probes on food packaging. ACS Nano 12(4):3287–3294. 10.1021/acsnano.7b08010 29621883 10.1021/acsnano.7b08010

[CR57] Zeng P, Chen X, Qin YR, Zhang YH, Wang XP, Wang JY, Ning ZX, Ruan QJ, Zhang YS (2019) Preparation and characterization of a novel colorimetric indicator film based on gelatin/polyvinyl alcohol incorporating mulberry anthocyanin extracts for monitoring fish freshness. Food Res Int 126:108604. 10.1016/j.foodres.2019.108604 31732021 10.1016/j.foodres.2019.108604

[CR58] Zhai X, Shi J, Zou X, Wang S, Jiang C, Zhang J, Huang X, Zhang W, Holmes M (2017) Novel colorimetric films based on starch/polyvinyl alcohol incorporated with roselle anthocyanins for fish freshness monitoring. Food Hydrocoll 1(69):308–317. 10.1016/j.foodhyd.2017.02.014 10.1016/j.foodhyd.2017.02.014

[CR59] Zhang J, Zou X, Zhai X, Huang XW, Jiang C, Holmes M (2019) Preparation of an intelligent pH film based on biodegradable polymers and roselle anthocyanins for monitoring pork freshness. Food Chem 272:306–312. 10.1016/j.foodchem.2018.08.041 30309548 10.1016/j.foodchem.2018.08.041

[CR60] Zhang KL, Huang TS, Yan H, Hu XZ, Ren T (2020) Novel pH-sensitive films based on starch/polyvinyl alcohol and food anthocyanins as a visual indicator of shrimp deterioration. Int J Biol Macromol 145:768–776. 10.1016/j.ijbiomac.2019.12.159 31866540 10.1016/j.ijbiomac.2019.12.159

[CR63] Zhao Y, Simonsen J, Cavender G, Jung J, Fuchigami LH (2019). U.S. Patent No. 10,334,863. Washington, DC: U.S. Patent and Trademark Office

